# Morbidity and mortality predictivity of nutritional assessment tools in the postoperative care unit

**DOI:** 10.1097/MD.0000000000005038

**Published:** 2016-10-07

**Authors:** Şule Özbilgin, Volkan Hancı, Dilek Ömür, Mücahit Özbilgin, Mine Tosun, Serhan Yurtlu, Semih Küçükgüçlü, Atalay Arkan

**Affiliations:** a Dokuz Eylul University, School of Medicine, Department of Anesthesiology and Reanimation; b Dokuz Eylul University, School of Medicine, Department of General Surgery; c Dokuz Eylül University, Faculty of Medicine, Postoperatif Care Unit, Izmir, Turkey.

**Keywords:** mortality, nutrition assessment, nutritional score, postoperative

## Abstract

The aim was to evaluate the nutritional situation of patients admitted to the Postoperative Acute Care Unit using classic methods of objective anthropometry, systemic evaluation methods, and Nutrition Risk in Critically Ill (NUTRIC) score, and to compare them as a predictor of morbidity and mortality.

At admission to the postoperative care unit, patients undergoing various surgeries were assessed for the following items: Subjective Global Assessment (SGA), Nutritional Risk Index (NRI), Nutritional Risk Screening (NRS)-2002, Mini Nutritional Assessment (MNA), Charlson comorbidity index (CCI), and NUTRIC score, anthropometric measurements, serum total protein, serum albumin, and lymphocyte count. Patients were monitored for postoperative complications until death or discharge. Correlation of complications with these parameters was also analyzed.

A total of 152 patients were included in the study. In this study a positive correlation was determined between mortality and NRS-2002, SGA, CCI, Acute Physiology and Chronic Health Evaluation , Sepsis-related Organ Failure Assessment, and NUTRIC score, whereas a negative correlation was determined between mortality and NRI. There was a correlation between NUTRIC score and pneumonia, development of atrial fibrillation, delirium, renal failure, inotrope use, and duration of mechanical ventilation. In our study group of postoperative patients, MNA had no predictive properties for any complication, whereas SGA had no predictive properties for any complications other than duration of hospital stay and mortality.

The NUTRIC score is an important indicator of mortality and morbidity in postoperative surgical patients. NRI correlated with many postoperative complications, and though SGA and NRS were correlated with mortality, they were not correlated with the majority of complications. MNA was determined not to have any correlation with any complication, mortality, and duration of hospital stay in our patient group.

## 1 Introduction

Malnutrition involves insufficiency developing in protein, energy, vitamin, and trace elements linked to insufficient and unbalanced nutrition, and is a tableau that increases the development of negative clinical events such as morbidity and mortality.^[1]^ Malnutrition is one of the independent factors with greatest effect on results after surgery, and lengthens hospital stay and affects mortality and morbidity.^[2,3]^ The prevalence of malnutrition in hospitalized patients is reported to be 20% to 42%.^[4]^ This rate is reported as 22% to 62% for patients undergoing gastrointestinal cancer surgery.^[5,6]^ In the geriatric patient population undergoing orthopedic surgery, there is an increase in risk of malnutrition.^[7]^

There are many tools created to define nutrition risk which use a variety of criteria.^[8,9]^ The majority of these indicators were not primarily designed for critical patients requiring acute care.^[10]^ There is no international consensus on a single “best tool.” The use of different tools in different studies has prevented comparison between studies and has not allowed definition of a “best tool“ for a certain patient population, age group, or environment.^[11]^ Especially, there are very few specifically designed screening tools to determine predictivity of clinical outcomes like morbidity, mortality, postoperative complications, or duration of hospital stay.^[11]^ Heyland et al^[12]^ first presented the new screening tool of Nutrition Risk in Critically Ill (NUTRIC) score in Europe. This tool was primarily developed as a nutritional risk assessment and was validated especially for intensive care patients. It is an easily and simply applied scoring system based on a few headings to determine malnourished patients.^[12]^ ASPEN 2016 Guideline suggests using the NUTRIC score.^[13]^

The hypothesis of our study is to evaluate the nutritional situation of patients admitted to the Postoperative Acute Care Unit (PACU) using classic methods of objective anthropometry, systemic evaluation methods, and NUTRIC score, and to compare them as a predictor of morbidity and mortality. To test this hypothesis, the aim of our research is to evaluate nutrition situations in cases admitted to the PACU using classical methods of objective anthropometry, Nutritional Risk Index (NRI), Nutritional Risk Screening (NRS-2002), Subjective Global Assessment (SGA), Mini Nutritional Assessment (MNA), Charlson comorbidity index (CCI), and NUTRIC score, and to determine its value as predictor of morbidity and mortality.

## 2 Materials and methods

The study is a prospective, descriptive, and cross-sectional study. Our study was completed after receiving permission from Dokuz Eylül University, Faculty of Medicine, Ethics Committee (Dokuz Eylul University School of Medicine Ethics Committee, date: 09/07/2015 and Dec. no.: 2094-GOA) and patients/their family provided written informed consent. Prospectively, within a 3-month period, general surgery, orthopedic, and urology cases above the age of 18 years treated in the PACU for more than 24 hours in the postoperative period and they were included in the study.

Inclusion criteria for the study were determined as patients with PACU admission after general surgery, and surgery related to orthopedics and urology. Cases below the age of 18 years, with psychiatric disorders, patients who were difficult to cooperate with, coma patients who could not give nutrition history, patients fed enterally or parenterally, with vomiting symptoms, taking appetite-enhancing medications, and pregnant or breastfeeding patients were excluded from the study.^[14–15]^

Consent was obtained 1 day before from the cases. Patients were not begun on additional parenteral or enteral products for nutritional situations; the nutritional regime for the patients’ clinical situation provided by their own physician was applied. We assessed nutritional status and laboratory parameters of nutrition in patients on admission to PACU (postoperative first day).

### 2.1 Evaluation of parameters

#### 2.1.1 Demographic data

Demographic data for all cases were recorded (age, sex, operation). Height was recorded from case notes where available or measured with a stadiometer. Weight was measured with bed measuring weight. Height and weight were used to determine body mass index (BMI) (weight [kg]/height [m^2^]). Weight change over the 6 months before hospital admission was estimated by patients and expressed as a percentage of previous weight.

#### 2.1.2 Assessment of biochemical parameters

Biochemical parameters including complete blood count, lymphocyte count, and serum levels of total protein, albumin, liver function tests, blood urea nitrogen (BUN), creatinine, and electrolyte measurements were taken within the postoperative first day. All biochemical parameters and all screening tools were determined on the same day as anthropometric evaluation by routine methods and classified according to reference values of the biochemistry laboratory

#### 2.1.3 Assessment of nutritional risk

##### 2.1.3.1 Anthropometric measurements

Measurements included triceps skin fold (TSF) and arm circumference (MAC). Anthropometric measurements were taken when the patient was admitted to the PACU by an independent physician. Three measurements were made to the nearest 0.1 cm. The mean of these measurements was recorded. TSF thickness (mm) was measured as follows: a calipers was used to measure the posterior face at the midpoint of the distance from the acromion and olecranon processes. TSF depth of 4 to 8 mm was the limit of fat deposition, whereas 3 mm or less was classified as severe loss. Upper mid-arm circumference (MAC) (cm) was calculated as follows: measured on the nondominant arm at the midpoint of a line joining the olecranon and acromion with the aid of a tape. MAMC (cm) = MAC (cm) − (TSF (mm) × 0.3412. MAC of 15 cm or less was accepted as severe loss of muscle mass, MAC of 16 to 19 cm was moderate loss, and MAC of 20 to 22 cm was evaluated as slight loss.^[16]^

##### 2.1.3.2 Subjective Global Assessment

This is a clinical score. It was performed by a trained independent physician using a standard form including food intake and complaints such as vomiting, diarrhea, and loss of weight. This information is used to classify patients into 1 of 3 categories of nutritional status: A—well nourished, B—moderately malnourished, or C—severely malnourished.^[17]^

##### 2.1.3.3 Nutritional Risk Index

This is a simple equation that uses serum albumin and recent weight loss. NRI (1.489 × serum albumin, g/L) + 41.7 × (present weight/usual weight). An NRI >100 indicates that the patient is not malnourished, 97.5 to 100 indicates mild malnourishment, 83.5 to <97.5 indicates moderate malnourishment, and <83.5 indicates severe malnourishment.^[18]^

##### 2.1.3.4 Nutritional Risk Screening

The NRS-2002 consists of a nutritional score and severity of disease score and an age adjustment for patients aged >70 years (+1). Nutritional score was calculated as follows: weight loss >5% in 3 months or food intake below 50% to 75% in preceding week = 1; weight loss >5% in 2 months or BMI 18.5 to 20.5 kg/m^2^ and impaired general condition or food intake 25% to 60% in preceding week = 2; and weight loss >5% in 1 month or >15% in 3 months or BMI <18.5 kg/m^2^ and impaired general condition or food intake 0% to 25% in preceding week = 3. Severity of disease score: hip fracture, chronic patients with acute complications = 1; major abdominal surgery, stroke, severe pneumonia, hematological malignancies = 2; and head injury, bone marrow transplantation, intensive care patients with Acute Physiology and Chronic Health Evaluation (APACHE) score >10 = 3. NRS-2002 score is the total of the nutritional score, and severity of disease score and age adjustment. Patients are classified as no risk = 0, low risk = 0 to 1, medium risk = 3 to 4, and high risk ≥45.^[19]^

##### 2.1.3.5 Mini Nutritional Assessment—Screening Form (MNA-SF)

The shorter form of MNA is a nutritional screening tool especially designed for the older population. It consists of 6 questions, scored from 0 to 2 or 3. These questions address present weight loss, appetite, mobility, psychological stress, neuropsychological problems, and BMI. Patients are categorized as having “normal nutritional status,” being at “nutritional risk” and “malnourished.”^[20]^

##### 2.1.3.6 Charlson comorbidity index

This index gives 1 point to all forms of coronary artery disease, in addition to congestive heart failure, peripheral vascular and cerebrovascular diseases, dementia, chronic pulmonary disease, connective tissue disease, peptic ulcer, mild liver disease, and diabetes. The score for hemiplagia and organ damage in addition to diabetes, any tumor, leukemia, and lymphoma is 2. Moderate or severe liver disease has a score of 3 and AIDS or metastatic solid tumor score is recorded as 6.^[21]^

##### 2.1.3.7 NUTRIC score

For each patient, the NUTRIC score was calculated using the patient's age, number of comorbidities, number of days between admittance to hospital and admittance to PACU, APACHE II at admittance,^[19]^ and Sequential Organ Failure Assessment (SOFA) score.^[20]^ NUTRIC points were calculated without interleukin (IL)-6; the tool's creators allow exclusion of this variable if not clinically available. As a result, patients with a total score of 5 or more were accepted as having high points and were classified as having high malnutrition risk.

#### 2.1.4 Assessment of complications

The presence, type, and severity of complications and mortality occurring after admittance were obtained from patient files after the patient was discharged. To avoid subjective observations, solid objective criteria were created to describe complications. Complications were defined as wound infection (inflammation/purulent discharge, positive swab culture), pneumonia (shadowing on new lung x-rays, purulent sputum ± positive culture, atelectasis [confirmed on CXR in the absence of signs of pulmonary infection]), pulmonary complications (pulmonary complications apart from pneumonia and atelectasis), sepsis (systemic inflammatory response syndrome criteria in addition to positive culture), intra-abdominal abscess (intra-abdominal purulent collections requiring operative drainage), cardiac arrhythmia (all types of arrhythmia not present before surgery), atrial fibrillation (irregular R-R interval [if there is AV conduction], recurring clear lack of P waves, irregular atrial activity, and variability in atrial cyclus length [rate of > 180 beats/min]), renal failure (<0.5 mL/kg/h urine discharge), delirium (neuropsychiatric symptoms and findings with acute onset and globally disrupting brain functions), and other (all other types of unexpected event requiring treatment or intervention). Morbidity data, and the duration of stay of patients in the PACU and in hospital were recorded and mortality on the 28th day was evaluated.

### 2.2 Statistical analysis

The SPSS 15.0 (Chicago, IL) program was used. Data with categorical values (BMI, TSF, MAMC, age, weight height) are presented as mean ± standard deviation (SD). To compare the anthropometric and systemic evaluation methods in the research, Mann–Whitney *U* test were used. Data indicating frequency are given as number and percentage (%). To compare malnutrition situation and frequency data, the chi-square test was used. To determine correlation, the Pearson correlation test was used. A *P* value <0.05 was accepted as a statistically significant difference.

## 3 Results

**Figure 1:**
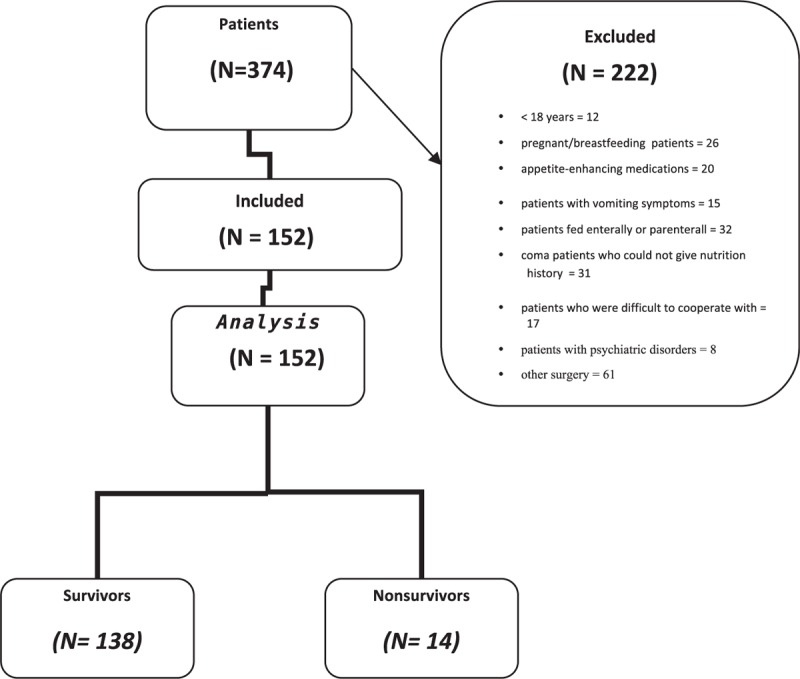
CONSORT flow diagram.

In all, 374 patients were included in this study. However, after the application of exclusion criteria, data of 152 patients were included in the analysis (Fig. 1). The patients included 78 (51.3%) general surgery, 62 (40.8%) orthopedic, and 12 (7.9%) patients from other clinics. Patients were operated on for the following: 74 major intra-abdominal interventions (48.7%), 54 major orthopedic interventions (35.5%), 10 other general surgery interventions (6.6%), 7 extremity surgery (4.6%), and 7 other surgical interventions (4.6%).

Malnutrition assessment found that according to NRS-2002 score, 30 patients (19.7%) had points below 3, whereas 122 patients (80.3%) had points of 3 and above. The SGA score found that 64 patients (42.1%) were well-nourished, whereas 74 patients (48.7%) were moderately malnourished and 14 patients (9.2%) were malnourished to an advanced degree. The MNA score evaluated 8 patients (5.2%) as having normal nutritional situation with points between 12 and 14, whereas 92 patients (61.8%) were assessed in the malnutrition risk group with points between 8 and 11, and 50 patients (32.9%) were evaluated as malnourished with points between 0 and 7. The NRI of patients found that 2 patients (1.3%) were not malnourished, 1 patient (0.7%) was slightly malnourished, 47 patients (30.9%) were moderately malnourished, and 102 patients (67.1%) were severely malnourished. When the patients were evaluated with the NUTRIC score, 118 patients (77.6%) had low score (between 0 and 4), whereas 34 patients (22.4%) were evaluated as having high score (between 5 and 9).

The anthropometric characteristics of patients and anthropometric values of deceased and surviving patients are given in Table 1. As the age of patients increased, there was a positive correlation with death (*r* = 0.214) and delirium (*r* = 0.188) (*P* < 0.05). There was a negative correlation between TSF values of patients and death (*r* = −0.182) (*P* < 0.05). The laboratory values of patients are shown in Table 2. There was a negative correlation between hemoglobin values and duration of hospital stay (*r* = −0.319), wound infection (*r* = −0.198), and delirium (*r* = −0.175) (*P* < 0.05). There was a negative correlation between hematocrit values and duration of hospital stay (*r* = −0.329), wound infection (*r* = −0.217), pulmonary complications (*r* = −0.168), and delirium (*r* = −0.180) (*P* < 0.05). Between platelet values and pneumonia (*r* = 0.307) and delirium (*r* = 0.193), there was a positive correlation (*P* < 0.05). There was a positive correlation between sodium values and sepsis (*r* = 0.266), and inotropic agent use (*r* = 0.233) (*P* < 0.05). Between calcium values and death (*r* = −0.244), pulmonary complications (*r* = −0.175), mechanical ventilation (*r* = −0.188), and inotropic agent use (*r* = −0.192), there was a negative correlation (*P* < 0.05). There was a positive correlation between aspartate transaminase **(**AST) values and mortality (*r* = 0.200), sepsis (*r* = 0.332), and inotropic agent use (*r* = 0.217) (*P* < 0.05). There was a positive correlation between glucose values and sepsis (*r* = 0.173) (*P* < 0.05). Between albumin values and duration of hospital stay (*r* = −0.298), mortality (*r* = −0.355), pulmonary complications (*r* = −0.257), sepsis (*r* = −0.191), and inotropic use (*r* = −0.246), there was a negative correlation (*P* < 0.05). There was a negative correlation between protein values and duration of hospital stay (*r* = −0.244), mortality (*r* = −0.226), sepsis (*r* = −0.184), and inotropic use (*r* = −0.247) (*P* < 0.05).

**Table 1:**
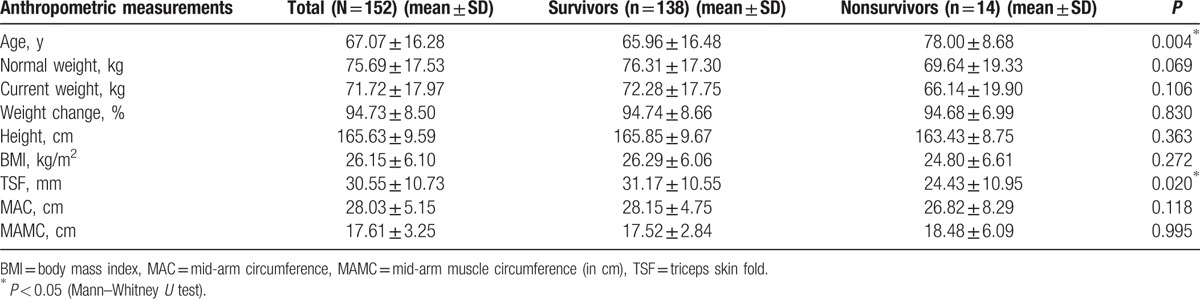
Anthropometric measurements of patients.

**Table 2:**
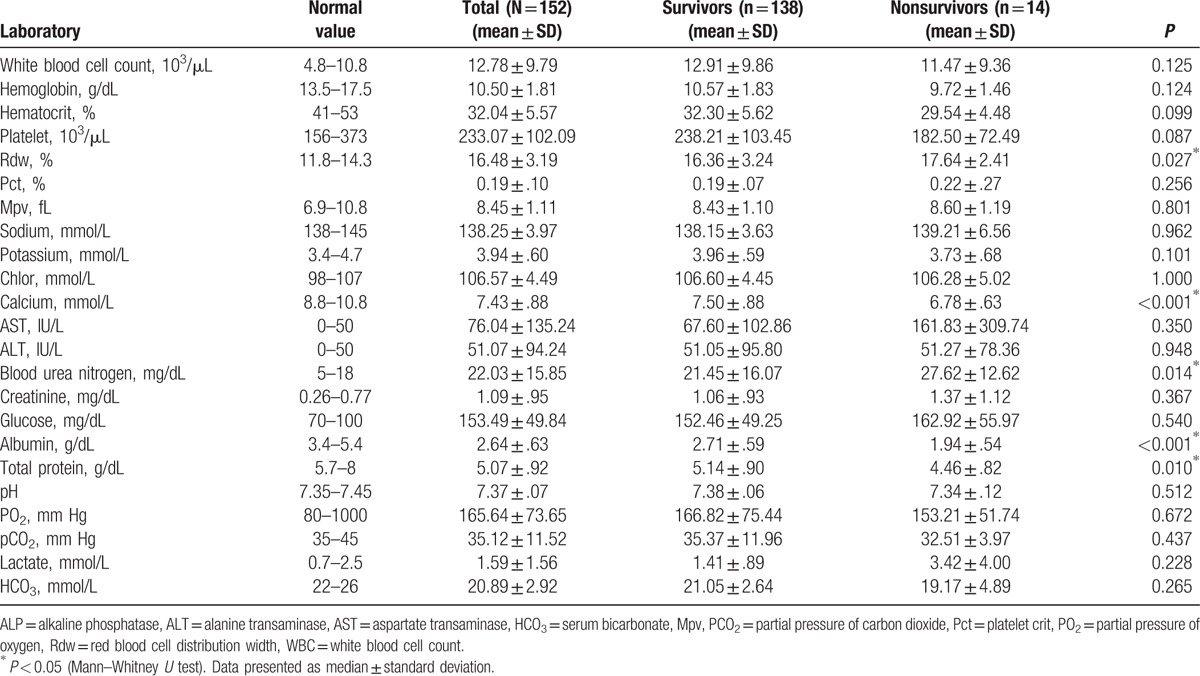
Laboratory measurements of patients.

In our study, in terms of mortality, there was a positive correlation with NRS-2002, SGA, CCI, APACHE, SOFA, and NUTRIC score, and a negative correlation with NRI. The nutritional risk score values and correlation between nutrition risk scores and complications of patients are shown in Tables 3 and 4.

**Table 3:**
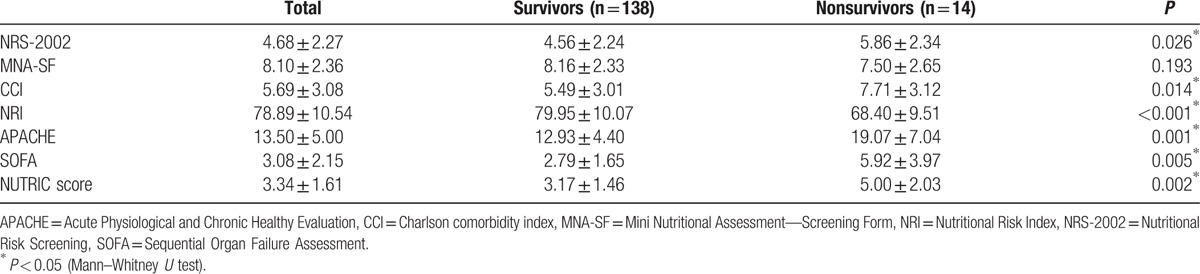
Values of Nutrition screening tools.

**Table 4:**
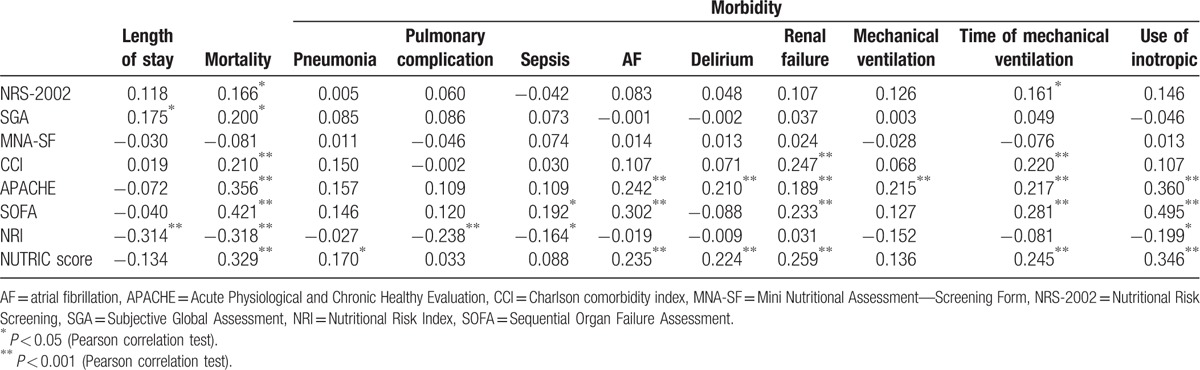
Correlation of nutrition screening tools with complications.

## 4 Discussion

This study determined a correlative relationship between patients NUTRIC score and mortality. The complications pneumonia, development of atrial fibrillation, delirium, renal failure, inotropic use, and mechanical ventilation duration in postoperative surgery cases treated in the PACU were also correlated with NUTRIC score. It was also determined that in our study patients in terms of mortality, there was a positive correlation with age, NRS-2002, SGA, CCI, APACHE, SOFA, and NUTRIC score, and a negative correlation with TSF and NRI. There was a relationship found between Ca, albumin, protein, lactate, and bicarbonate values, and developing complications and mortality.

A variety of methods are used to evaluate the nutritional situation of patients admitted to hospital. As there is no gold standard of nutritional evaluation, and as the majority of methods are inconvenient and time-consuming, they are not routinely used.^[11]^ SGA is an assessment tool based fully in clinical evaluation. Studies have found that mortality is higher in groups with malnutrition determined by SGA and anthropometric data. In our study, though there was a positive correlation between SGA and mortality, and duration of hospital stay, it is interesting that we did not find any correlative relationship between SGA and any complications in our patients.

There are studies showing that MNA is predictive of mortality.^[10]^ Persson et al^[22]^ showed that MNA had good predictivity for mortality estimation in the long term. Some MNA studies have shown that this screening tool is beneficial to determine clinical complication/adverse findings; however, it is not predictive of negative results.^[23,24]^ Results of a systematic review of nutrition screening tools in 2014 found that MNA may be predictive of mortality and hospital stay; however, there were no findings showing predictivity for complications in quality studies.^[25]^ In our study, similar to some previous studies,^[23,24]^ there was no correlative relationship between MNA and mortality. In our study, parallel to the meta-analysis results,^[24]^ there was no correlation between any of the complications screened in our study with MNA.

The CCI has been shown to be a good predictor of both short-term (hospital stay and the following 30 days) and long-term (12-month) mortality.^[26]^ Ather and Nazim^[26]^ evaluated the impact of CCI on overall survival after tumor nephrectomy, and identified that CCI had a significant predictive value on overall survival. Tokgöz et al^[27]^ showed patients with higher preoperative CCI scores may have higher postoperative complication rates and CPCS grades. They reported that after an radical nephrectomy operation, uro-oncologists must be alert to the symptoms and signs of postoperative complications in patients with chronic pulmonary diseases and high preoperative CCI scores. The same researchers attempted to identify the prognostic factors that might predict a worse outcome in nonsurvivors compared with survivors of Fournier gangrene, and observed that a high CCI was associated with high mortality.^[28]^ Similar to previous studies in our patients, comprising the postoperative surgical patient group, there was a significant correlation between CCI and mortality, renal failure, and mechanical ventilation duration.

The NUTRIC score is based on a conceptual model designed around how to measure acute and chronic hunger, and inflammation, especially under intensive care conditions.^[12]^ Studies have shown that in the intensive care unit (ICU) patient population, acute hunger, chronic hunger, and inflammation predictors are effective on nutritional situation and patient results.^[12]^ When validating this score, a relationship was shown in both discrimination between heterogeneous ICU patients, and this risk score and results. In later times, a variety of studies were completed to evaluate malnutrition and results in the critical patient group requiring aggressive nutrition treatment using this score.^[29–32]^ In NUTRIC score history, like measurement of reduced nutrition, reduced oral intake, or weight loss, is not an effective factor. This score can correctly identify patients with high mortality rates or survivors with longer durations of hospital stay. Heyland et al^[12]^ started by considering the need for a more specific nutritional risk evaluation tool for ICU patients, and found that inquiring about weight loss and nutritional situation was insufficient due to the heterogeneous nature of the intensive care population especially, and as the NUTRIC score has easy-to-use characteristics, they stated it was an important screening tool for this patient group. They demonstrated that patients with a higher score have worse clinical outcomes. They considered greater awareness of nutrition risk assessment tools, such as the NUTRIC score, and risk factors, such as BMI and duration of ICU stay, may enhance the delivery of calories and protein to those patients who need these the most. Coltman et al^[29]^ showed traditional screening and assessment tools did not uniformly identify patients as malnourished or at nutrition risk in the ICU and therefore may be inappropriate for use in ICU patients. Inclusion of physical assessment, functional status, and severity of illness may be useful in predicting nutrition risk in the ICU. The patient group in our study, comprising the postoperative surgical patient population, was similar to previous studies.^[29–32]^ Whereas there was a correlation between NUTRIC score and mortality, there was no correlation found with duration of hospital stay. Additionally, in our study, there was a correlative relationship between NUTRIC score and pneumonia, atrial fibrillation, delirium, renal failure, mechanical ventilation duration, and inotropic use. In this way, NUTRIC score was predictive of more complications that many of the malnutrition markers evaluated in our study.

The results of this study should be interpreted with the fact that these results are derived from a single center's PACU patients, and the overall number of patients included in the analysis is quite low. A multicentric prospective study with higher patient inclusion would support these findings. However, none of the tools used alone may be sufficient for correct prediction of all result measurements (duration of stay, mortality, complications) in all patient groups, in all circumstances, and in all age groups. Studies comparing tools for a single patient population have found very small differences between these tools and the other tools used in these studies. Our recommendation is that to screen or evaluate the nutritional situation of patients, a single tool should never be fully trusted. When compared with reference methods in different studies, all tools may show low diagnostic accuracy and none of the tools may show good predictive validity for all result measurements. As a result, clinical decisions always continue to play an important role. Screening and evaluation tools may be applied as the first step in nutritional screening all the time; however, it is necessary that users be aware of what limitations apply to the tools.^[33–35]^

In conclusion, NUTRIC score is an important indicator of mortality and morbidity in postoperative surgical patients. NRI correlated with many postoperative complications, and though SGA and NRS were correlated with mortality, they were not correlated with the majority of complications. MNA was determined not to have any correlation with any complication, mortality, and duration of hospital stay in our patient group.
